# The Role of Non-Caloric Sweeteners in Sensory Characteristics of Pastry Products

**DOI:** 10.3390/foods8080329

**Published:** 2019-08-08

**Authors:** Vilma Quitral, Juanita Valdés, Valeska Umaña, Nicol Gallardo, María José Alcaino, Carolina Araya, Marcos Flores

**Affiliations:** 1 Escuela de Nutrición y Dietética, Facultad de Salud, Universidad Santo Tomas, Ejercito 146, Santiago 8370003, Chile; 2 Departamento de Ciencias Básicas, Facultad de Ciencias, Universidad Santo Tomas, Avenida Carlos Schorr 255, Talca 3473620, Chile

**Keywords:** non-caloric sweeteners, sensory characteristics, physical parameters, useful life

## Abstract

The purpose of this work was to evaluate the role of non-caloric sweeteners in pastry products considering the product made with sugar as a control sample. Sensory preference and acceptability with consumers were determined through ranking test and 9-point hedonic scale respectively. The satiation and satiety were determined by the visual analogue scale (VAS). In addition, caloric intake; macronutrients; physical parameters such as yield, specific volume, hardness, cohesiveness, and gum; stability in storage time by microbiological analysis; and moisture variation of the samples were calculated. The results showed that the preference and sensory acceptability is significantly (*p* < 0.05) higher in the control sample; the sweeteners decreased the sensory response, but not the satiation and satiety, although these are not related to the sensory response and did not present significant differences with the control sample, except in the satiation parameter of the sample sweetened with Stevia. The physical and texture parameters highlight the best quality of the control sample and are related to the sensory response. The shelf life is also greater in the control sample, which shows that sugar contributes sweetness and other technological characteristics related to texture, stability during storage, aroma, color, and flavor, thanks to the Maillard reaction.

## 1. Introduction

The consumption of sugary foods contributes to a high amount of calories for the human being, so sugar replacement is a desirable strategy to apply in different foods [1]. In the case of foods that have been baked, sugar (sucrose) on the one hand fulfills the function of sweetening baked-food products, in addition to other technological roles which are difficult to achieve with non-caloric sweeteners, such as stevia, polyols, or a combination of them; the roles of sugar are: effect on food texture, mouth feel, color of food thanks to the Maillard reaction, and moisture stabilization among other properties [2,3].

Today, to reduce the amount of sugar in processed foods, strategies have been sought that allow formulating sweet foods with sweeteners without or with very low caloric intake [4,5,6,7,8]. On the other hand, there are natural and synthetic sweeteners that are incorporated as ingredients alone or in mixtures [9,10]. Therefore, foods sweetened with non-caloric sweeteners provide lower caloric content and may be preferred by consumers concerned about consuming fewer calories. However, the sensory quality of food can be affected, in addition to other characteristics such as humidity, texture and shelf life [11,12].

Non-caloric sweeteners must be approved by specialized agencies, such as the Joint FAO/WHO Expert Committee on Food Additives (JEFCA), the European Food Safety Authority (EFSA), and the Food and Drug Administration (FDA), that evaluate their safety and risks. This evaluation establishes an acceptable daily intake (ADI), which according to Joint FAO/WHO, is defined as “amount of a food additive that for a human, expressed on the basis of body weight, can be consumed daily even during all of life, without risk”.

To compare the sweetening effect of sweeteners, the response of a sucrose solution of standardized concentration and conditions (sucrose solution 30 g/L at 20 °C) is taken as reference, to which the value of 1 is assigned [13,14,15].

Among non-caloric sweeteners that are commonly used in processed foods are stevia, sucralose, tagatose, and polyols.

Stevia is a sweetener of natural origin and is extracted from a plant in the province of Misiones, Paraguay. The natives of the area called it “sweet grass” and used it as a healing medicine. The Paraguayan chemist Ovidio Rebaudi managed to isolate the active principles responsible for the sweetness of the plant in about 1900. Stevioside and rebaudioside are two of the sweet glycosides in the leaves of the shrub; stevioside consists of a molecule of steviol in which the lower hydrogen atom is replaced by a β-d-glucose molecule, and the upper hydrogen is replaced by two molecules of β-d-glucose. Healthy effects are attributed to it as antihypertensive and antihyperglycemic [16]. Its caloric intake is 2.7 kcal/g and its relative sweetening power is 300 times compared to sucrose. It presents an adequate thermal stability, for this reason it is suitable for baked foods [14]. Stevia-rebaudioside A (Rbd-A) is the second most abundant stesviol glycoside found in the leaves of Stevia rebaudiana Bertoni; it is the main sweet constituent of the Rebaudiosides family [17]. BESTEVIA^®^ Reb M stevia leaf sweetener is 200 to 300 times sweeter than sugar. The process starts with the leaf of the stevia plant and results in a 95% pure Reb M stevia sweetener with significantly less bitterness and aftertaste than conventional stevia sweeteners. This enables you to achieve levels of sugar reduction that were impossible or unpalatable until now [18].

On the other hand, tagatose is a monosaccharide of six carbons with a ketone group that belongs to the ketohexosas group, an epimer of D-fructose isomerised at C-4. Tagatose is accepted and recognized as safe by the FAO/WHO since 2001. It can be found naturally in very low concentrations in fruits and dairy products and is produced commercially from the lactose molecule. It can also be obtained by bioconversion from D-galactose whey [19]. It has prebiotic character, since it is fermented by the microflora of the colon and produces short chain fatty acids; its structure corresponds to a ketohexose and is similar to D-fructose in its structure and in its metabolism, but it is not completely absorbed; a consequence of this type of structure is that it can suffer from the non-enzymatic browning reaction (Maillard reaction). It has the advantage that it is not hygroscopic, and its solubility is similar to sugar; with other sweeteners it has a synergistic effect, so it is very convenient to combine it with other sweeteners. Its caloric intake is 1.5 kcal/g, with a glycemic index equal to zero, and its relative sweetness is 0.92 [14,15].

Sucralose is an artificial sweetener, obtained by the selective halogenation of the sucrose molecule, in which three atoms of chloride are substituted in hydroxyl groups. However, most of the ingested sucralose is not absorbed in the gastrointestinal tract, but is excreted directly in the stool. It has a relative sweetness power of 600. It is very soluble in water and is stable over a wide range of temperatures and pH. If it is stored at high temperatures, it releases HCl and can produce some type of discoloration in food because of oxidation reactions, and it can also modify the pH of the medium [14,15,20].

Polyols, which are hydrogenated carbohydrates used as sugar substitutes, due to their sweet taste and low caloric power, can also be used alone or mixed with other sweeteners. Its absorption in the small intestine varies from 2 to 90%, and the non-absorbed remnant is fermented by the microflora of the colon. Among the polyols most commonly used in foods as sweeteners are sorbitol, xylitol, maltitol, isomalt, lactitol, mannitol, and erythritol. The caloric intake is different for each type of polyol, the lowest being for Erythritol (0.2 kcal/100 g) and the highest for sorbitol (2.6 kcal/g). The relative sweetness of maltitol and isomaltitol is 0.8–0.9 and 0.45–0.6 respectively, and the energy contribution is 3.0 and 2.0 kcal/g in each [14]. This type of compound must be added in low concentration in foods since they produce flatulence and diarrhea [21].

Other non-caloric or low calorie sweeteners used in food are Acesulfame-K, allulose, aspartame (it is stable when it is dry or frozen, but it decomposes and loses its sweetening power over time, when stored in liquids at temperatures above 30 °C), and Luo han guo extracts (fruit extract grown in China and Thailand, which is 300 times sweeter than sugar) [22].

In many foods, non-caloric or low-calorie sweeteners are used, mainly in soft drinks, fruits nectars, juices, yoghurts, fermented milks, chewing gum, and candies. They are also used, but to a lesser extent, in beverages with mineral salts, canned fruits and vegetables, breakfast cereals, ready-to-eat meals, sauces, condiments, and vegetable drinks [23].

Food intake reduces hunger; consumption of some of them has a greater effect, as well as has different periods of duration. Satiation evaluates the feeling of hunger or fullness that occurs during the meal; it is a process involved in the decision to end the food intake. Satiety measures that sensation after a certain time has elapsed since the ingestion. In both cases, there are sensory, cognitive, digestive, and hormonal signals [24]. Different analytical methods are used to evaluate satiation and satiety. This parameter can be measured through the quantification of glucagon, peptide YY, ghrelin, glucose, and insulin concentrations. Another alternative to evaluate this parameter is to weigh the amount of food consumed. It is also possible to apply the Visual Analogue Scale (VAS), which measures subjective sensations as indicators of regulation. It consists of a scale that starts in “I am very hungry” to “I am very satisfied”. These scales are completed before and after the meal, at regular intervals [25].

There are studies that have related satiety with the sensory response. It has been claimed that when the taste for a meal increases, feelings of satiation and satiety increase; however, others studies have found no differences in hunger and fullness ratings with the palatability of food, which shows that we have a controversial issue where there is a lack of knowledge [26]. Foods that are sensory well qualified, possibly generate preparatory physiological responses, such as the release of satiety hormones. Sensory signals can also activate cognitive processes, such as memory, and cause satiety [27].

The objectives of the present study are to evaluate the role of non-caloric sweeteners in sensory characteristics and physical parameters, and furthermore the satiation, satiety, and shelf life of these cupcakes.

## 2. Materials and Methods

### 2.1. Materials

Four types of cupcakes were prepared. The basic formula includes white flour, eggs, milk, butter, baking powder, and different sweeteners (sugar and non-caloric sweeteners (NCS) based in stevia, sucralose, and tagatose; doses are according to the recommendations delivered in each container). Formulations are presented in Table 1. Baking time was 55 min at 165 °C.

### 2.2. Sensory Analysis

Application of two sensory tests to assess the subjective response of consumers.

Ranking test was performed to evaluate the preference within the cupcakes with 55 subjects aged between 18 and 40 years old. Each subject received a tray containing four samples of cupcakes in dishes marked with random three-digit codes, a glass of water and response sheet. The sensory analysis was carried out according to the standard UNE-ISO 8587 [28].

Furthermore, the acceptability test applied was the 9-points hedonic verbal scale, from “dislike extremely” to “like extremely” [29]. One-hundred-and-sixty subjects aged between 18 and 40 years old, evaluated one sample each session. After the evaluation, each response of the hedonic scale was numbered from 1 to 9, where 1 corresponds to “dislike extremely” and 9 to “like extremely”.

### 2.3. Satiation and Satiety Evaluation

Visual analogue scale (VAS) were used to evaluate the subjective appetite sensation of the four cupcakes samples, with 160 subjects aged between 18 and 40 years, evaluating one sample each session. They consist of a scale, with 7 points, from “I’m very hungry” to “I’m very full”. VAS was applied before eating 50 g of a cupcake, immediately after consuming it (15 min approximately) and after 120 min of consumption. The second response corresponds to satiation, and after 120 min is satiety [25].

Analysis of variance (ANOVA) was carried out on the numbers of the scale, and Tukey’s pairwise multiple comparison tests (α = 0.05) were carried out. The averages of the VAS responses were plotted over time, equaling the baseline value.

### 2.4. Energy and Nutrients

An analytical method was carried out to determine total sugars [30]. Macronutrients were determined based on the nutritional information of the ingredients. Table 1 shows the quantities of each component used in the formulation of the different cupcakes.

### 2.5. Physical Analysis

Process yield was determined by weighting the raw batter and baked product and expressed as (%) of initial sample weight.

The cupcakes’ specific volumes were measured following the rapeseed displacement method, expressed as (cm^3^/g) [31].

Textural profile analysis (TPA) test was performed using Zwick/Roell Texture with 500 N load cell. Each sample for analysis was 2 × 2.5 × 2.5 cm, which is compressed twice consecutively, using a flat base cylindrical punch 25 mm in diameter, evaluating the hardness. Four repetitions were performed per sample. Textural parameters including Hardness (g), (the Hardness value is the peak force that occurs during the first compression), Cohesivity (is how well the product withstands a second deformation relative to its resistance under the first deformation, calculated from the area of work during the second compression divided by the area of work during the first compression), and Gumminess (g) (g) (Hardness × Cohesiveness).

### 2.6. Humidity

Thermogravimetric method was applied for size humidity in the storage time. According to 93,401 Official Methods of Analysis of Association of Official Analytical Chemists International. 16th Ed. AOAC, 1995, USA [32]. The samples were finely ground and approximately 5 g were weighed in dry crucibles previously weighed in analytical balance. The samples were placed in an oven at 105 °C for 4 h, after that time, they were removed from the stove with tweezers and placed in a desiccator for 30 min. Then the crucible was weighed in analytical balance. The process is repeated until a constant weight is reached. The humidity is calculated with the following formula:

$$\%\mathrm{humidity}\;=\frac{\left[B-C\right]}{\left(B-A\right)}\times 100$$

where: A = crucible weight, B = crucible weight plus sample, C = crucible weight plus dry sample.

### 2.7. Microbiological Analysis

Yeast and mold presence were detected in the samples in the storage time. Ten grams of each sample were weighed and homogenized with 90 mL of peptonated water solution. Dilutions were made and seeded in sterile plates. Potato dextrose agar was added, and the plates were incubated at 25 ± 1 °C for 3 to 5 days. The presence of yeast and mold colonies was observed.

### 2.8. Statistical Analysis

Analysis of variance (ANOVA) was carried out on the numbers of the scale, and Tukey’s pairwise multiple comparison tests (α = 0.05) were carried out.

All analyses were carried out using SPSS version 19.0 software.

## 3. Results

### 3.1. Sensory Analysis 

The ranking test results in a significantly greater preference for sample C0 (*p* < 0.05), see Table 2, placing it as the first preference; second place was C1; no significant difference was found with C2; the least preference was for C3; and no significant difference was found with C2.

The acceptability test, with 9-point hedonic scale, indicates that C0 has the highest acceptability, with a rating of 8, which is equivalent to “I like very much” with a significant difference (*p* < 0.05) with respect to the other samples.

**Figure 1:**
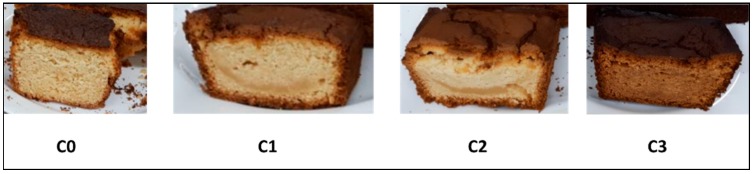
Photos of cupcake samples. Where different samples are C0 = based in Sugar; C1 = based in Stevia, Maltitol and Polydextrose; C2 = based in Sucralose, Maltitol and Polydextrose; C3 = based in Tagatose, Isomaltitol and Soluble Fiber.

Figure 1 shows the products made based on the different formulations proposed in this study.

### 3.2. Satiation and Satiety Evaluation

**Figure 2:**
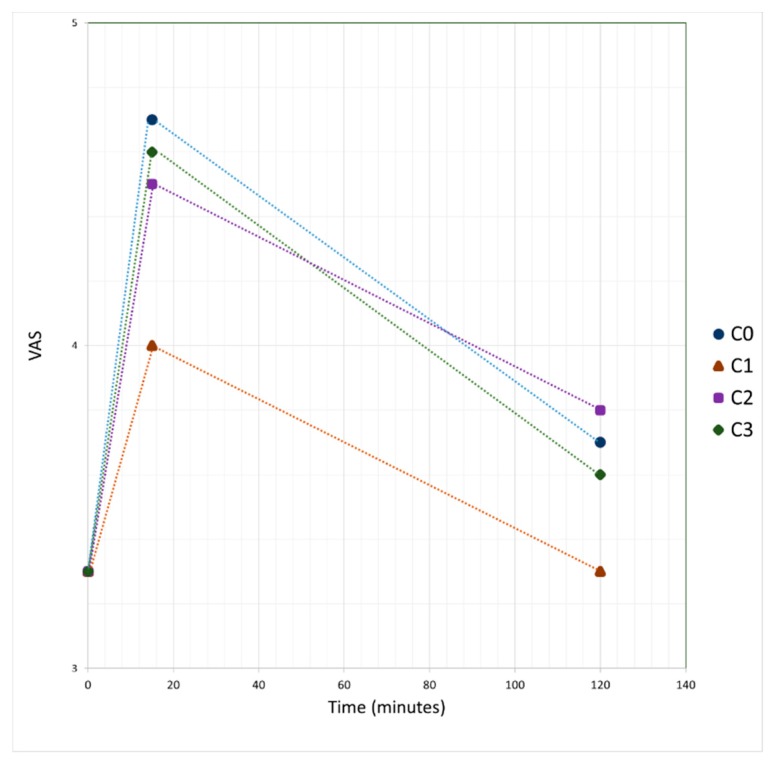
Satiation and satiety rating through time, before intake and after 15 and 120 min of intake of cupcake samples.

It is observed in Figure 2 that the satiation produced by the consumption of the samples is greater with C0, without significant differences between C3 and C2, while there is a significant difference with sample C1. As for satiety, the largest is produced by sample C2, and the smallest is C1, although the differences are not significant.

### 3.3. Energy and Nutrients

With respect to energy intake, carbohydrate and sugar are significantly higher in C0, since it was made with sugar, see Table 3. The NCS contained different polyols and polydextrose, so the content of total sugars in them is different, being the lowest in C2. The content of proteins and lipids varies due to the performance that is different between the samples.

### 3.4. Physical Analysis

Table 4 presents the physical properties of the samples; it is seen that there is no significant difference in the yield and cohesiveness between the different samples, despite the fact that the yield is higher in sample C2, as well as the cohesiveness is the lowest for the same sample; these differences are not significant.

### 3.5. Humidity

**Figure 3:**
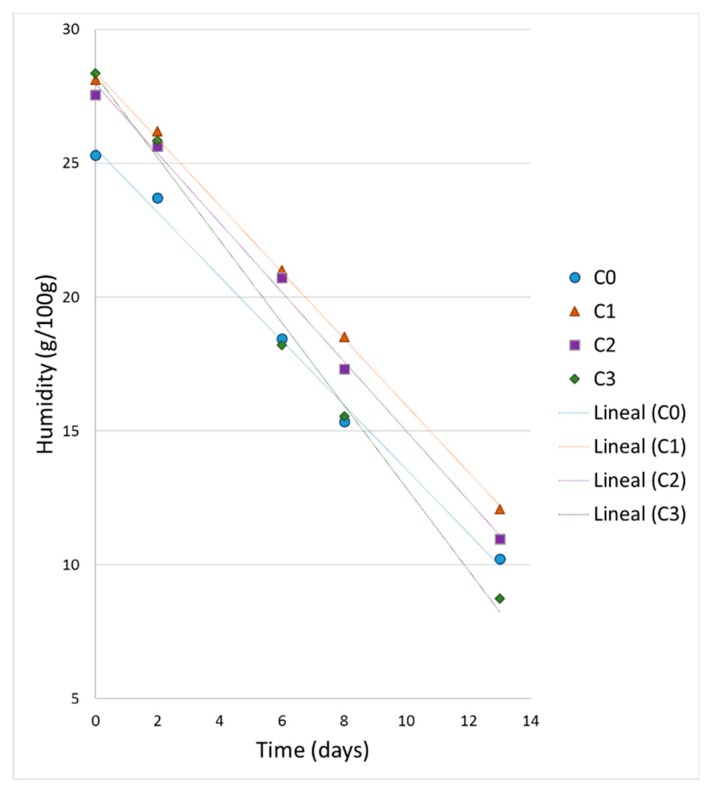
Moisture variation of cupcake samples over time.

Figure 3 shows a significant decrease in humidity over time. Sample C0 contains less moisture at the initial time, but over time it reaches similar values to samples C2 and C3. C0 contains sucrose, which has an effect on moisture retention [33,34]. Sample C1 has the highest humidity at the beginning of the storage period, however, it ends with the lowest humidity between the samples. On the other hand, C3 sample shows the greatest decrease in humidity over time.

### 3.6. Microbiological Analysis

The results indicate that the C0 sample presents more microbiological stability, see Table 5, since at day 25 it is still without the development of microorganisms. The sample with the lowest microbiological stability is C2, since at day 17 it presents microbial development.

## 4. Discussion

The color of the four cupcakes was different, see Figure 1, which directly affected the preference and acceptability. Sample C0 presents a more attractive and typical color, with toasted crust and yellow crumb; it also presents better aroma, flavor and texture than the other samples. C1, C2 and C3 presents few pores, they are crushed, and C1 and C2 are pale tonality, while C3 is very dark, in crumb and crust.

The C0 sample presents a bright bark of golden-brown and is very attractive, which coincides with previous results [35,36]. This characteristic color is the product of the Maillard reaction and the caramelization process. The Maillard reaction is produced by the interaction between reducing sugars and amino acids, and the caramelization comprises various reactions produced by high temperatures during baking on sucrose and reducing sugars [37]. The change in color is also affected when the sugar content in the preparation of muffins is reduced [33].

Gao et al. (2017) replaced sugar with stevia (50% and 100%) in the preparation of muffins, which directly affected the color (paler preparations with Stevia) [34]. The sensory evaluation performed in this study with a subjective response using a linear scale for different parameters did not show significant differences between muffins with sugar and 50% replacement with Stevia. However, replacing 100% with Stevia, the texture was classified as “harder”, visually less attractive, and generally less liked by the evaluators; these differences were significant.

The replacement of sugar by maltitol or by polydextrose significantly affects the sensory quality of cakes [36], a phenomenon that also occurred with samples C1 and C2 that contain this type of additives as part of the sweetener.

The flavor is also affected in the preparation of food by replacing sugar with another type of sweetener, as confirmed by other researchers [38]. In the study presented by Quitral et al. (2017), the taste of samples sweetened with sugar were better graded followed by samples with sweetened stevia and finally those sweetened with sucralose (*p* < 0.05), which coincides with the results of the present study [35].

Samples C1 and C2 in addition to stevia and sucralose, contain maltitol and polydextrose, the latter component has shown good results, which is attributable to the fact that it is formed by glucose molecules, which is why the Maillard reaction suffers as a result of thermal degradation, favoring the color, aroma and taste of baked doughs [38]. However, this replacement is discussion matter since Ronda et al. (2005) affirmed that the replacement of sugar by maltitol or polydextrose significantly affects the sensory quality of cakes, since they present a strange and remaining taste, affecting acceptability [36].

Martinez-Cervera et al. (2012) showed that no differences were found in the general acceptability, appearance, color, texture, flavor, and sweetness between muffins made with sucrose and with 50% polydextrose replacement; however, by completely replacing sucrose with polydextrose, all the parameters evaluated by means of a 9-point hedonic scale are significantly reduced [33].

Maillard reaction is desirable from the sensory point of view, since it produces the characteristic color of baked foods, pleasant flavors and aromas; however, it affects the nutritional quality since the availability of some amino acids that participate in the reaction and with it the protein digestibility is lost. Additionally, some compounds declared as carcinogenic can be generated, such as acrylamide and hydroxymethylfurfural (HMF). On the other hand, the Maillard reaction produces melanoidins, which are attributed antioxidant (protecting food from lipid oxidation), antimicrobial and prebiotic activity [37,39,40,41].

De Souza et al. (2013) consider stevia as the best non-caloric sweetener, because it decreases the bitter taste generated by other sweeteners [42]. On the other hand, the natural extract of stevia has a characteristic taste described as “pure sweet” that is masked with the use of mixtures of other sweetening substances; these combinations of ingredients are frequently made by the food industry to avoid the bitter taste of the sweeteners.

The cupcake sweetened with tagatose was the least preferred by the evaluators, which coincides with a study conducted by Rubio-Arraez et al. (2018), where the sensory acceptability of a commercial gelatin sample (with sugar), one with 50% isomaltulose and 50% tagatose and another with 100% tagatose was compared; the sample with total sugar substitution by tagatose was the worst evaluated [43]. Sample C3 in this study (sweetened with tagatose) was classified as cloying, with a bitter taste or with a strange taste; this is contradicted by a study carried out by Mogha et al. (2016), in which they demonstrated that the sensory contribution of tagatose is beneficial, since it reduces the bitter aftertaste of the preparations significantly [44]. As the tagatose undergoes the Maillard reaction since it is a six-carbon monosaccharide with a ketone group, the color of the C3 sample was darker, both in the crust and in the crumb, and the strange taste can be attributed to the products of this reaction, which was higher than in the case of C0.

In the present study it was observed that the cake with the greatest acceptability was sweetened with sugar, in both men and women, which coincides with the results of the study by Martínez-Cervera et al. (2012), in which they replaced total or partial sucrose (25%, 50%, 75%) in muffins; the authors demonstrated that the 100% sucrose replacement cake obtained a lower acceptance than the control sample (with sucrose) [33]. This is due to the fact that the sugar, in addition to adding flavor to the food, adds beneficial physical qualities such as viscosity and consistency, as well as taste, aroma and texture, being characteristics that influence food preference [45].

In this study, C0 is the best sample and within samples with sweeteners, C1 is preferred, according to the results obtained by the ranking test. The preference test uses ordinal scales where numbers are used to know the order of preference of the products, but not the degree of preference that is generated between each of the products, while the acceptance test allows to identify the level of liking of each sample [46]. It is therefore important to measure both tests to ensure the quality of a food before being released to the market.

Sugar generates satiety since, being composed of glucose, it stimulates the release of insulin, which produces an anorectic effect by inhibiting the NPY-producing neurons [47]. Its absorption is rapid, but it has slower gastric emptying when compared to sucralose [48,49].

Previous studies show that sucralose does not alter the absorption of glucose or GLP-1 function and, therefore, neither the control of hunger and satiety. However, a recent study shows that sucralose produces an insulin response similar to that of sucrose [50]. Thus, causing a similar level of satiety, which is proven in the present study.

Tagatose has a prebiotic effect, since bacteria in the colon ferments it. Due to the above, its excessive consumption (more than 30 g per day) can cause flatulence and gastrointestinal alterations, but this does not affect the absorption of nutrients [51]. It has been shown that tagatose has the ability to stimulate the secretion of GLP-1 to a greater extent than sucralose, therefore, it would cause greater satiety. The results of the present study showed the opposite; C2 (with sucralose) produced greater satiety than C3 and C0 (with tagatose and sugar respectively), however, they are not statistically significant differences. The sweetener of sample C3 contains tagatose, isomaltitol and soluble fiber within its ingredients, so it would have been expected to produce greater satiation and satiety, due to the presence of fiber [52,53,54].

Stevia is characterized by having a flavor very similar to sugar, as well as having multiple health benefits, such as antibacterial, antiviral, anti-inflammatory, and regulation of blood sugar and blood pressure [55]. One study showed that individuals who consumed stevia did not compensate for eating more at the next meal and had similar levels of satiety compared to those who consumed sucrose; in addition, it lowered plasma glucose and insulin levels [49]. The above, could explain why stevia has produced less satiation, since, probably by decreasing insulin levels, its anorexigenic effect would not be manifested.

The edulcorant no caloric (ENC) effect has been evaluated in satiety using VAS, showing that they do not increase the sensation of hunger; in addition, its administration produces neutral or minimal effects in most of the intestinal hormones involved in the sensation of satiety [56]. It has even been proposed that ENC does not allow the release of satiety-inducing peptides [44,57]. However, more recent evidence indicates that ENCs do participate in the regulation of incretin systems such as the release of GLP-1 [58]. On the other hand, it is believed that they cause a dissociation in the sensation of sweet taste, which together with insufficient calories would cause a lower sensation of satiety, causing an increase in energy consumption due to an increase in appetite [59]. However, our results and those of other studies have shown that satiety is the same when consuming products made with sugar or with ENC; in this sense, they would participate in the regulation mechanisms of hunger satiety [56,58].

When relating satiety with sensory quality, no correlations were found for samples sweetened with sugar, sucralose and tagatose (C0, C2 and C3 respectively). In samples sweetened with stevia, a negative correlation was found, to better evaluations in the hedonic scale, lower satiety (R = 0.7885); which is contradictory with the idea that sensory factors would influence in satiation [60].

Our results are partially opposed to the study proposed by Mattes and Vickers (2018), where they applied a questionnaire of satiety of five factors to relate to the sensory effect on satiety; these authors concluded that when eating foods that are enjoyed, much more pleasure, satisfaction and satiety are experienced [61]. However, if a food is sensorially very pleasant, this sensation occurs at the beginning; if you continue having the pleasant sensation while eating, you will even stop eating because the sensation of pleasure is minimal, rather than feeling satiated [62].

When comparing our values with the literature, these are lower than those determined by Marchetti et al. (2018) in muffins, in which all the samples have a yield greater than 85 g/100 g; additionally, they are similar to the yields obtained by Zhang et al. (2018) in the preparation of bread, where there was a variation of the molecular weights of dextran used in the samples studied [63,64]. The yield represents the loss of water (steam) during baking and is associated with the interactions between the matrix and the water.

In the case of the specific volume of the baked products, milk and egg proteins contribute mainly (Matos et al., 2014); these are present in all the samples in the same concentration, so the variation in this case can be attributed to the different sweeteners used in each sample.

The volume reached by baked doughs and a typical structure with many pores gives them the spongy texture, which is very characteristic and pleasant for consumption, therefore a high value of specific volume is an indicator of product quality [65].

The specific volume is significantly higher in the C0 sample, which indicates a higher final volume with greater gas retention capacity in the gluten network [33]. In the study by Ronda et al. (2005), where sugar in cakes was replaced by polyols and other sweeteners, the control sample also showed a higher specific volume [36]. The authors attribute this effect to the fact that sugar-free cakes do not produce the same expansion because they decrease the stability and viscosity of the dough during the heating stage. These results are consistent with the present study, since the replacement of sugar in the production of cupcakes with mixtures of maltitol plus polydextrose with sweeteners sucralose and Stevia, the specific volume is significantly lower. These results are consistent with the present study, since the replacement of sugar in the production of cupcakes with mixtures of maltitol plus polydextrose with sweeteners sucralose and Stevia, the specific volume is significantly lower.

Gao et al. (2017) replaced sugar with stevia (50% and 100%) in the preparation of muffins, and the samples showed lower volume at a higher Stevia content; in the samples where amount and pores size varied, firmness significantly increased and elasticity decreased when replaced by 100% Stevia [34].

Sample C3 has the lowest specific volume, it must be considered that this sample does not contain malititol. On the other hand, it has been reported that maltitol produces less adverse effects in bakery products when it is replaced by sugar, with respect to viscosity and volume of the product [66].

The sample C0 elaborated with sugar presents more pores than the rest of the other samples, since the latter are seen as compact and crushed; this can be attributed to the fact that the sugar delays the gelatinization temperature of the starch and protein denaturation during the baking of the dough, therefore there is an increase in volume and bubbles air bubbles are formed that expand due to the vapor pressure caused in the cooking process where they reach greater stability [21,34,36,37,38].

Hardness and gumminess texture variables increase significantly in C1, C2 and C3 with respect to C0, while cohesiveness increases in C1 and decreases in C2, without significant differences.

As for the hardness variable, sample C1 has the lowest value, at the same time as the highest specific volume, which can be attributed to the formation of a greater number of air bubbles that are stable; this increases the volume and gives sponginess to the product [21,34,36,38].

The results presented by sample C1 coincide with some researchers, who have shown that stevia causes increased hardness and cohesiveness in bakery products [37,67]. However, Manisha et al. (2012) found opposite results where a decrease of hardness and cohesiveness occurs when replacing sugar with Stevia and liquid sorbitol in cakes [68]. In this sense, it is necessary to carry out further research.

In the literature, it is demonstrated that sugar replacement by polydextrose causes a decrease in hardness by 40% in the preparation of muffins, and maltitol also significantly decreases hardness in muffins [36]. Contrary to our results where C1 and C2 samples containing polydextrose and maltitol present the highest values of hardness.

Larsen et al. (2016) argued that foods in which different types of texture are perceived by chewing them would affect satiation, since more sensory stimuli would be produced by each chew [69].

It has been established that foods with greater hardness increase the time of oral processing, activity of the muscles, and jaw movements; however, it has not been related to effects on satiety [47].

Regarding cohesiveness, it has been shown that Maltitol and isomaltitol cause a decrease in cohesiveness when replacing sugar in muffin making [21]. However, there are no significant differences between the results of our samples. Like polydextrose, present in samples C1 and C2, they do not cause a decrease in cohesiveness, as demonstrated in the study in muffins [36]. C0 contains sucrose, which has an effect on moisture retention [33,34].

The products of the Maillard reaction are attributed antimicrobial effects, which agrees with these results, since C0 was elaborated with sugar which favors the production of the Maillard reaction. C3 was elaborated with tagatose that also suffers a Maillard reaction; however, from the microbiological point of view, C3 reacted as well as the C1 sample that was made with stevia.

The shelf life of the cupcakes was greater when they were made with sugar, since sugar is an agent that helps moisture stability, limiting the swelling of the starch, resulting in a finer final texture. The volume of the cakes made with sugar is also greater, since it improves the aggregation of fat crystals and thus captures and retains air during the shake, which coincides with the results of texture, in which the dough is softer and less gummy.

The reduction of sugar in bakery products is complex; reductions of 15–20% give good results, maintaining the different properties of the food, but higher percentages of substitution imply the use of mixtures of sweeteners and the incorporation of polyols and fiber [14].

On the other hand, sweeteners due to their composition and high sweetening power should be used in minimum quantities, which is not always easy to determine, otherwise it generates undesirable changes in the preparations as bitter or residual taste [69], which makes evaluators tend to prefer traditionally-sweetened products.

## 5. Conclusions

According to the results, it is concluded that the effect of replacing 100% sugar with non-nutritive sweeteners affects bakery products from a sensory, physical and vital point of view. According to the ranking of preference and acceptability, the cakes prepared with sugar maintain a significant difference with respect to non-caloric sweeteners. While satiety is not affected for the different preparations, there are differences in microbiological activity; on the one hand, the effect of sugar does not allow microbial growth during the entire study, however, non-caloric sweeteners do allow it from day 17. On the other hand, when considering the non-nutritive sweeteners used based on stevia, sucralose and tagatose, and considering the different criteria evaluated in this study, the best response is presented by C1 sample, sweetened with stevia. 

## Figures and Tables

**Table 1:** Formulations of cupcakes samples.

	Samples			
	C0	C1	C2	C3
White flour (g)	100	100	100	100
Eggs (units)	1	1	1	1
Milk (mL)	80	80	80	80
Butter (g)	50	50	50	50
Baking powder (g)	10	10	10	10
Sugar (g)	95	0	0	0
Stevia, maltitol, polydextrose (g)	0	47.5	0	0
Sucralose, maltitol, polydextrose (g)	0	0	47.5	0
Tagatose, isomaltitol, soluble fiber (g)	0	0	0	47.5

**Table 2:** Preference and acceptability ranking of cupcake samples.

Sample	Preference	9-Point Hedonic Scale Acceptability (Average)
C0	1° ^a^	8 ^a^ = I like very much
C1	2° ^b^	7 ^b^ = I like moderately
C2	3° ^bc^	7 ^b^ = I like moderately
C3	4° ^c^	7 ^b^ = I like moderately

Different letters indicate significant differences (*p* < 0.05).

**Table 3:** Nutritional composition of cupcake samples per 100 g.

Samples	C0	C1	C2	C3
Energy (kcal)	399 ^b^	315 ^a^	292 ^a^	298 ^a^
Proteins (g)	6.4 ^a^	7.3 ^b^	6.8 ^ab^	7.0 ^ab^
Total lipid (g)	15.4 ^a^	17.6 ^c^	16.3 ^ab^	16.8 ^bc^
Carbohydrate (g)	58.6 ^b^	31.8 ^a^	29.6 ^a^	29.6 ^a^
Sugars, total (g)	30.8 ^c^	4.7 ^a^	2.8 ^a^	13.2 ^b^

Different letters indicate significant differences (*p* < 0.05).

**Table 4:** Physical and textural characteristics of cupcake simples.

Samples	Yield (g/100 g)	Specific Volume (cm^3^/g)	Hardness N	Cohesiveness	Gum gf
C0	80.0 ^a^	6.5 ^a^	8.3 ^a^	0.26 ^a^	208 ^a^
C1	80.1 ^a^	3.5 ^b^	25.8 ^b^	0.27 ^a^	704 ^b^
C2	86.7 ^a^	3.2 ^b^	29.5 ^b^	0.24 ^a^	723 ^b^
C3	84.0 ^a^	2.9 ^b^	23.3 ^b^	0.26 ^a^	624 ^b^

Different letters in each column indicate significant differences (*p* < 0.05) among the samples.

**Table 5:** Mold and yeast count in cupcake samples.

	Samples			
Time (Days)	C0	C1	C2	C3
3	−	−	−	−
8	−	−	−	−
14	−	−	−	−
17	−	−	+	−
25	−	+	+	+
